# Imu-based kinematic analysis to enhance upper limb motor function assessment in neuromuscular diseases

**DOI:** 10.1186/s12984-025-01602-2

**Published:** 2025-03-18

**Authors:** Alessandra Favata, Roger Gallart-Agut, Luc van Noort, Jesica Exposito-Escudero, Julita Medina-Cantillo, Carme Torras, Daniel Natera-de Benito, Josep M. Font-Llagunes, Rosa Pàmies-Vilà

**Affiliations:** 1 Department of Mechanical Engineering and Institute for Research and Innovation in Health (IRIS), Universitat Politècnica de Catalunya - BarcelonaTech (UPC), Barcelona, Spain; 2 Institut de Recerca Sant Joan de Déu, Santa Rosa 39-57, 08950 Esplugues de Llobregat, Spain; 3 Institut de Robòtica i Informàtica Industrial, CSIC-UPC, Llorens i Artigas 4-6, 08028 Barcelona, Spain; 4 Neuromuscular Unit, Department of Neurology, Hospital Sant Joan de Déu, Barcelona, Spain; 5 Applied Research in Neuromuscular Diseases, Institut de Recerca Sant Joan de Déu, Barcelona, Spain; 6 Department of Rehabilitation, Hospital Sant Joan de Déu, Barcelona, Spain

**Keywords:** Neuromuscular diseases, Upper limb kinematic, IMU-based system, PUL, RULM

## Abstract

Duchenne muscular dystrophy (DMD) and spinal muscular atrophy (SMA) are neuromuscular diseases that lead to progressive muscle degeneration and weakness. Recent therapeutic advances for DMD and SMA highlight the need for accurate clinical evaluation. Traditionally, motor function of the upper limbs is assessed using motor function scales. However, these scales are influenced by clinician’s interpretation and may lack accuracy. For this reason, clinicians are becoming interested in finding alternative solutions. In this context, Inertial Measurement Units (IMUs) have gained popularity, offering the possibility to quantitatively and objectively analyze motor function of patients to support clinicians’ assessments. We analyzed upper limb kinematics of two groups of children with neuromuscular diseases, seventeen DMD patients and fifteen SMA patients, while performing the corresponding clinical assessment. These two groups were further subdivided into two categories (Category A and Category B), according to disease severity (Brooke scores $\le 2$ and Brooke scores $>2$, respectively). The results were compared against a group of ten healthy children. The metrics showing the strongest correlation with the clinical score were the workspace area in the frontal and transverse plane (DMD: $\rho$ = 0.94 and $\rho$ = 0.90; SMA: $\rho$ = 0.78 and $\rho$ = 0.81) and the workspace volume (DMD: $\rho$ = 0.92; SMA $\rho$ = 0.81). Additionally, statistically significant differences were found not only between healthy children and those with neuromuscular disease, but also across severity levels within the patient group. These results represent a first step toward validating IMU-based systems to helping clinicians to accurately quantify the motor status of children with neuromuscular diseases. Furthermore, data collected with inertial sensors can provide clinicians with additional information not available through subjective observation.

## Introduction

Neuromuscular diseases (NMDs) affect the peripheral nervous system, the muscle, or the neuromuscular junction. Muscle weakness is the primary symptom shared by all neuromuscular conditions [1]. Traditionally, lower-limb impairment has received great attention. Nevertheless, new therapeutic advances have increased the interest in preserving quality of life and upper-limb function. Additionally, these therapeutic advances of treatment and management of NMDs highlight the need for accurate and reliable clinical outcome metrics. Such metrics are not only essential in the monitoring of disease progression but also for evaluating the efficacy of new therapies [1–4]. Traditionally, the assessment of motor function in neuromuscular diseases relies on observer-dependent motor scales, typically administered during hospital visits.

In conditions like Duchenne Muscular Dystrophy (DMD) and Spinal Muscular Atrophy (SMA), two of the most common neuromuscular diseases in children, upper limb motor function is assessed using disease-specific scales: the Performance of Upper Limb (PUL) scale for DMD and the Revised Upper Limb Module (RULM) scale for SMA [5, 6]. Both contain a list of items describing movement tasks to perform. Based on the performance of the movement task, a clinician assigns a score for each item (usually either 0, 1 or 2 points). While these functional motor scales are considered the gold standard for monitoring meaningful clinical changes, they often lack sensitivity in detecting subtle motor function changes characteristic of slowly progressive diseases and are dependent on clinician’s subjective interpretation, which may introduce variability into the assessment [7–9]. Additionally, their points-based scoring system lacks the resolution needed to assess upper limb kinematics accurately.

In recent years, the limitations of these traditional assessment methods have prompted research into more refined and objective evaluation methods. One such method is the instrumented kinematic analysis using inertial measurement units (IMUs). IMUs offer a means of capturing detailed and quantitative data on movement and motor function. These objective data can enhance the accuracy and reliability of clinical assessments, providing valuable insights that complement the evaluations of specialists [10].

Multiple studies have focused on instrumented quantification of disability in DMD or SMA. Armand et al. [11] and D’Angelo et al. [12] performed a gait analysis in children with DMD and SMA, underlying the importance of providing additional information to clinicians to enhance therapeutic decision-making. Matsumaru et al.[13] developed two indices to evaluate the upper limb kinematics of patients with SMA using a single retro-reflective marker, with the disadvantages of remaining bounded to a motion analysis laboratory. In [3, 4, 14–17], the authors performed an accurate evaluation of upper limb kinematics suggesting metrics to monitor disease progression, but did not focus the attention on instrumenting the clinical assessment. Panero et al. [18] reviewed studies using IMU-based systems to assess meaningful outcome measures for children with DMD. Interestingly, the authors highlighted the lack of reliable and accurate measures for upper extremity in DMD patients. For these reasons, this study focuses on the use of an IMU-based system to assess the upper limb kinematics in both DMD and SMA patients, while performing the standard clinical assessment.

In this study, we evaluated an IMU-based system with seven IMUs, in a clinical setting with a group of patients with DMD, SMA and a group of healthy controls. The goal of this study was to determine whether the IMU-based system was capable of providing useful, quantitative, and precise outcome metrics with the potential to inform clinical decision-making. We hypothesized that this system will provide objective outcomes that correlate with the scores assigned during the clinical assessments, while offering additional insight beyond what is captured by traditional evaluations.

## Materials and methods

### Subjects

Twenty children with DMD, nineteen children with SMA, and eleven healthy children participated in this study (Table 1). Eight children were excluded from the analysis due to sensor-related technical issues. Specifically, in six cases, a sensor detached during recording due to adhesive patch failure. In the remaining two cases, one sensor failed to record data correctly. Inclusion criteria for the DMD and SMA groups were: (1) a confirmed diagnosis of DMD or SMA, and (2) an age range between 6 and 18 years old. For the healthy group, the inclusion criterion was an age range between 6 and 18 years old, and the exclusion criterion the presence of any disease affecting the mobility of the upper or lower body parts.

The study received approval from the Ethical Committee of the Hospital Sant Joan de Déu (Esplugues de Llobregat, Barcelona, Spain; approval number: PS-28-22). All parents or legal guardians of the participants signed the written informed consent form.

**Table 1:** Demographic information of the analyzed participants after the exclusion of a total of eight children due to technical issues

Type	n	Gender*	Age	Ambulant(%)	Type
DMD	17	17/17	12.4 ± 2.7	30	
SMA	15	11/15	10.1 ± 2.8	0	3 SMAII ; 12 SMAIII
Healthy	10	9/10	10.3 ± 4.2	100	

*number of males/total participants. Age is shown as mean± SD, Type of disease was applicable just for children with SMA

### Equipment

**Fig. 1:**
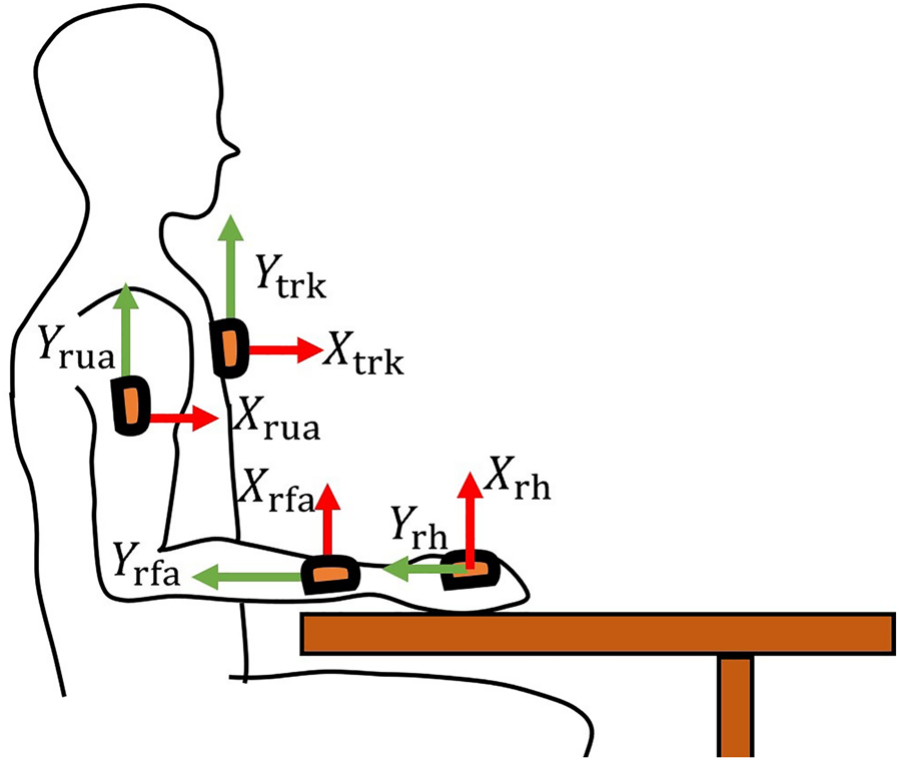
Sensors placement: on the torso at the sternum level, on the proximal part of both upper arms, on the distal part of both forearms at the wrist level, and on the dorsal surface of both hands

The data collection procedure was conducted at the Hospital Sant Joan de Déu (Esplugues de Llobregat, Barcelona, Spain). Upper limb kinematics was recorded with an IMU-based system with 7 sensors placed on the torso, at sternum level, and one on each upper arm, forearm, and hand (see Fig. 1). The IMUs used in the study were Xsens DOT sensors (Xsens Technologies, Netherlands). Each Xsens DOT is a compact wearable device (size: 36.3 x 30.4 x 10.8 mm (l x w x h); weight: 11.2 g) containing a 3D accelerometer, gyroscope, and magnetometer, which measure the linear acceleration, angular velocity, and earth magnetic field, respectively. Combined with the Xsens Kalman Filter core (XKFCore) sensor fusion algorithm, embedded in the sensor firmware, the sensor’s 3D orientation was provided. The sampling frequency was 60 Hz [19]. Data output, in the form of unit quaternions, was imported into MATLAB (MATLAB R2023b, The MathWorks, USA), where kinematics was computed. The sensors were attached using adhesive patches to ensure stability.

### Functional upper limb clinical scales

Functional assessments were performed using the PUL 2.0 scale for individuals with DMD, and the RULM scale for individuals with SMA. Both assessments were conducted while participants wore the IMU-based system. The PUL 2.0, a validated tool for both late-ambulant and non-ambulant DMD patients, comprises 22 items [20]. Similarly, the RULM is validated for functional motor assessment in SMA patients and comprises 20 items [6]. Each item was graded either 0 (unable), 1 (completed independently but with modification), or 2 (completed without compensation). The total score for each assessment is given by the sum of the individual score assigned to each item. The total score for the PUL ranges between 0 and 42, while for RULM ranges between 0 and 37. A higher score indicated better upper limb functionality.

Both scales start with the same entry item. This item coincides with the Brooke scale (see Table 2) [21]. This scale ranges from 1 to 6, where 1 indicates normal simultaneous arm abduction, and 6 reflects no useful hand function. The clinician begins by asking the patient to abduct both arms to ear level without elbow flexion; if successful, a score of 1 is assigned, otherwise 2. If arm abduction is not possible, the patient is asked to lift a cup to their mouth, scoring 3 if successful and 4 if not. If only hand movement or no useful hand function is possible, scores of 5 or 6 are given, respectively. Based on the Brooke score, some items of the PUL may be skipped as indicated by the clinical scale. In this study, the PUL and RULM are performed with both hands. Participants performed the same item twice, first with the dominant hand and then with the other.

### Kinematic model

A segmental biomechanical model was used to compute the upper limb kinematics. This model assumes three rotational degrees of freedom considering each joint as spherical joint, without translational components. Specifically, we considered the humerus articulating with the thorax, omitting scapular movement relative to both the thorax and humerus [22, 23]. We identified seven body segments: trunk (trk), right upper arm (rua), left upper arm (lua), right forearm (rfa), left forearm (lfa), right hand (rh) and left hand (lh), as shown in Fig. 1. The coordinate system was constructed with an inferior-superior directed Y-axis, a posterior-anterior X-axis, and a medial-lateral Z-axis, as suggested by the standard [22]. Regarding the joint angles, for the shoulder we computed the angle of the plane of elevation together with the elevation and axial rotations, while for the elbow and wrist we computed the flexion/extension, adduction/abduction, and internal/external rotations [16, 22, 24].

For each child, we measured the distance between the two acromia for the shoulder, the distance between the acromion and the lateral epicondyle for the upper arm, the distance between the lateral epicondyle and the ulnar styloid for the forearm, and the distance between the proximal carpal bones and the third metacarpal for the hand. We used these measurements to personalize the kinematic model for each child. Joint positions were computed by combining segment lengths with their respective absolute 3D orientations.

### Sensor-to-segment calibration

The quaternions provided by the sensors represent the orientation of the sensor frame in the East-North-Up coordinate system. This sensor coordinate system is defined as follow: X-axis positive to the East, Y-axis positive to the North and Z-axis positive when pointed up [19]. Therefore, a calibration was needed to establish the relative orientation between the sensor’s coordinate system and the one of the body segment to which it is attached [25]. Common calibration methods are: assumed alignment (AA), functional alignment (FA) and augmented data (AD) [26]. Each method has its own advantages and disadvantages. The first method consists of attaching the sensor while trying to align as best as possible the coordinate system of the sensor with the coordinate system of the body segment, but it heavily relies on the expertise of the clinicians. FA requires the patients to perform dynamic movement(s) but patients with NMDs might not be able to perform it or the evaluator has to help the patients. Finally, the AD requires additional tools and may not be feasible in a clinical environment. Thus, common calibration methods were not adequate to be applied in children with neuromuscular diseases [23, 26]. For this reason, we decided to work with a picture-based method for sensor-to-segment calibration [27, 28]. We asked participants to perform such calibration with their torso straight and forearm lying on a table. Then, we took three pictures (right and left lateral planes and frontal plane), while the subject was performing this pose. We used the positions of various anatomical landmarks obtained through image analysis, to determine the 3D relative orientation between the body segments and the sensors’ coordinate systems. The selected anatomical landmarks (i.e., processus xiphoideus, acromion, medial and lateral epicondyle, ulnar and radial styloid, and the third metacarpal) were manually extracted from three images. By combining the coordinates of these landmarks, the 3D relative orientations between body segments were computed and subsequently used to calibrate the sensors. Additionally, a scaling factor was applied to minimize discrepancies caused by variations in the camera-to-participant distance across the images.

### Data analysis and statistics

**Fig. 2:**
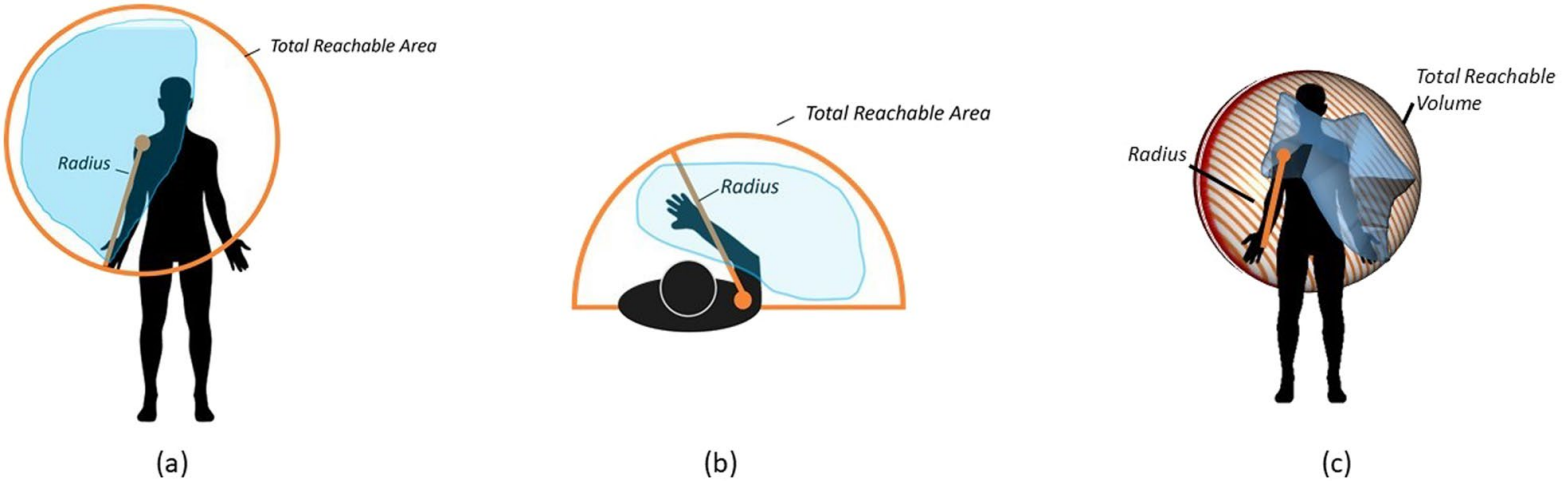
Representation of the workspace area in the frontal **a** and transverse **b** planes and workspace volume **c**. The orange envelope marks the total reachable area/volume with the radius equal to the sum of the lengths of the upper arm, forearm and hand. The blue shaded area provides an example of the workspace area that was reached

The following metrics were evaluated in accordance with relevant literature [3, 4, 16, 29]: Frontal and transverse normalized workspace area, normalized workspace volume (Fig. 2), curve efficiency, shoulder and elbow range of motion (ROM), and hand linear velocity. These metrics were obtained while the children were performing the items, manually annotating the items on the videos. To offer a deeper understanding of the motor status of the children, we compared the workspace area reached for the segmented recording (annotated data, corresponding to movements performed during clinical assessment) and during the full recording (without annotation). This approach enabled us to evaluate the motor status of the participants, beyond the constraints of clinical assessments. We also specifically analyzed the entry item, as it determines the Brooke score for both scales (see Table 2). Furthermore, we also compared the workspace area of the dominant and non-dominant sides.

**Table 2:** Detailed description of the brooke scale

Description	No useful function of hands	Can use hands to hold pen, pick up pennies from table	Can raise hands to the mouth, but cannot raise a 8-oz glass of water to the mouth	Cannot raise hands above head, but can raise a 8-oz glass of water to the mouth	Can raise arms above head only by flexing the elbow or using accessory muscles	Starting with arms at the sides, the patient can abduct the arms in a full circle until they touch
Brooke score	6	5	4	3	2	1

We defined the **normalized workspace area** as the area of the two dimensional envelope, obtained with the Delaunay triangulation method, around all positions of the hands in both the frontal and transverse planes [30]. Each area was normalized with respect to each subject’s maximum achievable area. In the frontal plane, the achievable area was defined as the disk with the shoulder joint as its center and the length of the arm as its radius (Fig. 2a). In the horizontal plane, it was defined as half of this disk (Fig. 2b). We hypothesized that a positive correlation would be identified between normalized workspace area and the clinical score.

We obtained the **normalized volume** for each child as the volume bounded by the 3D convex hull of the hand’s position (Fig. 2c). It was normalized with respect to the maximum achievable volume, defined as half of the sphere considering the shoulder joint as its center and the length of the arm as its radius.

We defined the shoulder-elbow **curve efficiency** as the sum of the shoulder elevation range of motion (ROM) and elbow flexion ROM divided by the number of samples that the movement spans [29]. If the movement was supposed to be performed using only the shoulder (i.e., for PUL items 1 and 2, and RULM item O), elbow flexion ROM was instead subtracted from shoulder elevation ROM to obtain the curve efficiency. See Appendix A for more information regarding the items. Curve efficiency was calculated for each item and the mean of all items was obtained. This metric was evaluated as a measure of interjoint coordination, where higher values represent more efficient movement.

We calculated the shoulder and elbow **ranges of motion (ROM)** as the difference between the maximum and the minimum of each joint angle recorded across all the items of the clinical scale.

We obtained **wrist linear velocity** assuming the same segments lengths for all participants (mean lengths for each segment of the selected cohort subset) expressed in the coordinate system of the torso.

Correlations between these metrics and PUL/RULM clinical scores were analyzed using the Spearman coefficient ($\rho$) [4, 15]. The correlation was interpreted as ’low’ if $\rho <0.45$, ’fair’ when $0.45<\rho <0.75$, and ’strong’ when $\rho>0.75$. For correlation calculations, only participants with DMD or SMA were considered. Healthy participants were not considered for computing correlation to prevent potential misinterpretation of the Spearman’s coefficients, even though they were included in graphs as reference.

Subject results were stratified according to the Brooke score, where children with neuromuscular diseases were categorized into two groups: Category A (Brooke scores $\le$ 2) and Category B (Brooke scores > 2), according to their ability to lift their arm above the head or not, respectively [15]. This classification allows us to address the issue of the limited number of participants in each Brooke category, while enabling us to evaluate the effectiveness of the IMU-based system in assessing a clinically meaningful function, such as lifting the arm overhead. Normality of each dataset was assessed using the Shapiro-Wilk test. Category differences were evaluated based on the Kruskal-Wallis test or ANOVA, followed by the Dunn-Sidak or Tukey-Kramer test as *post-hoc* approach according to data distribution. Within-group comparison was performed with a paired t-test or a Wilcoxon signed-rank test, as appropriate. Statistical significance was set at $p < 0.05$, corrected for multiple comparisons when appropriate. All statistical analyses were conducted using IBM SPSS Statistics 30.0.0. (IBM, NY, USA).

## Results

We will report the kinematic data of a total of 17 children with DMD, 15 children with SMA, and 10 healthy children since three children with DMD, four with SMA and one healthy child were excluded from the study due to system failure and/or sensor detachment during recording. Over the 10 healthy children analyzed in this study, 6 performed the PUL and 4 the RULM. The results obtained will be reported over two sections. Section 3.1 will present an overview of the kinematic analysis of participants with DMD as well as healthy controls that performed the PUL. Section 3.2 will contain kinematic analysis results for participants with SMA and for those controls that performed the RULM.

**Fig. 3:**
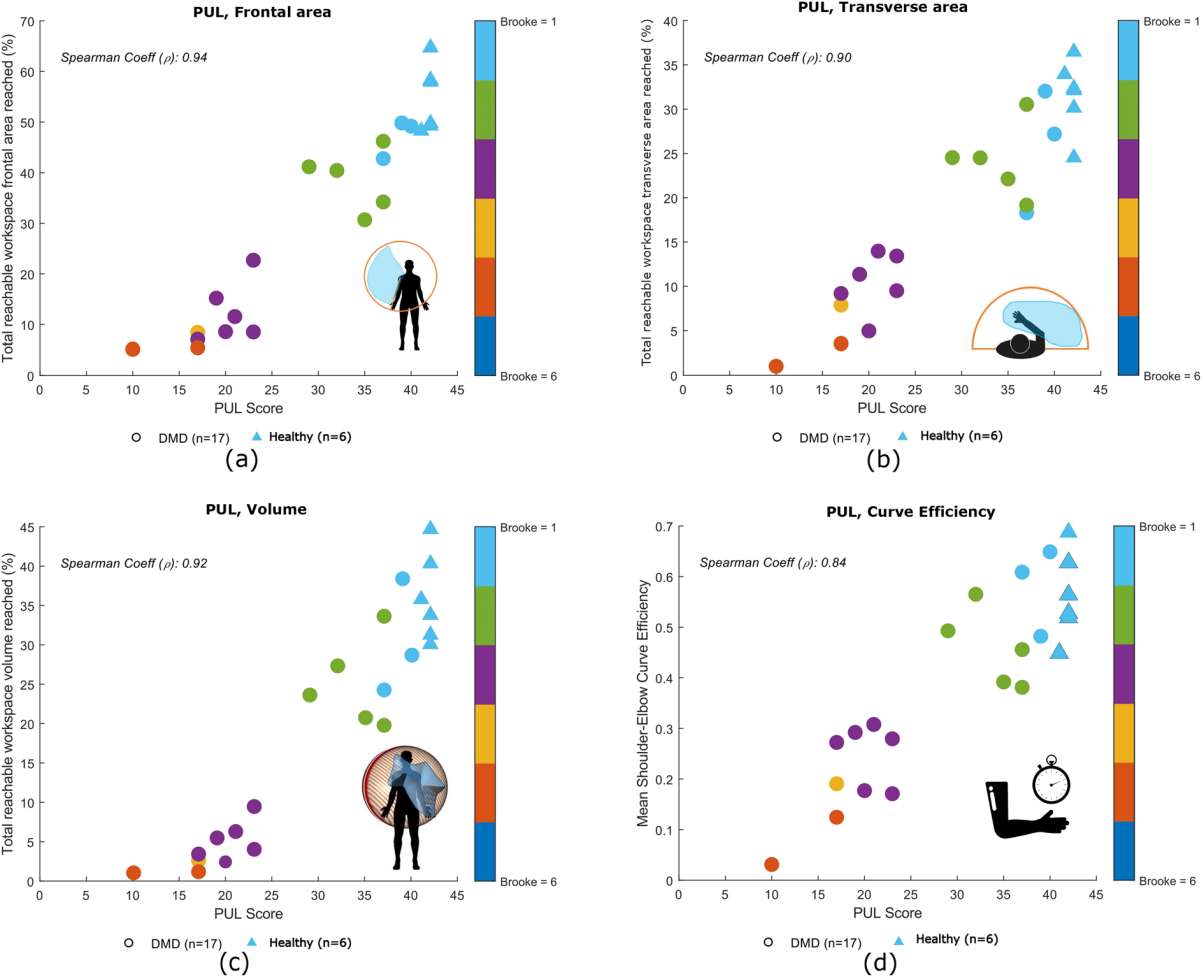
Correlation plots for children with DMD (PUL clinical scale): **a** correlation between clinical score and workspace area in the frontal plane, **b** correlation between clinical score and workspace area in the transverse plane, **c** correlation between clinical score and workspace volume and **d** correlation between clinical score and curve efficiency. Spearman correlation coefficient $\rho$ between the metric and the clinical score is reported. Healthy children are not included in the correlation analysis

**Fig. 4:**
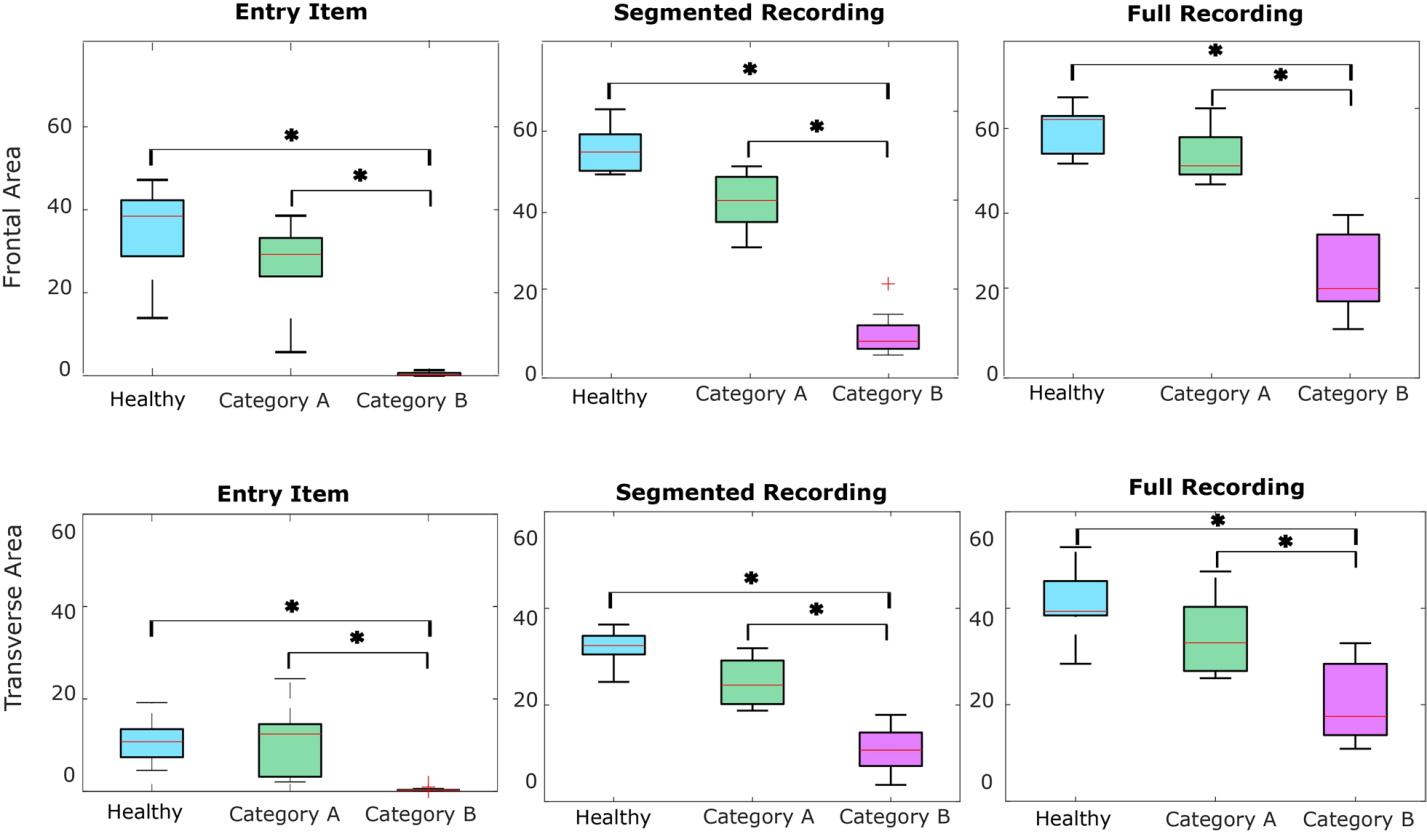
Boxplots of the workspace area of children with DMD in the frontal plane (upper row) and transverse plane (lower row) for the entry item, for all the item after the annotation and without annotation. Statistically significant differences are denoted with an asterisks

**Fig. 5:**
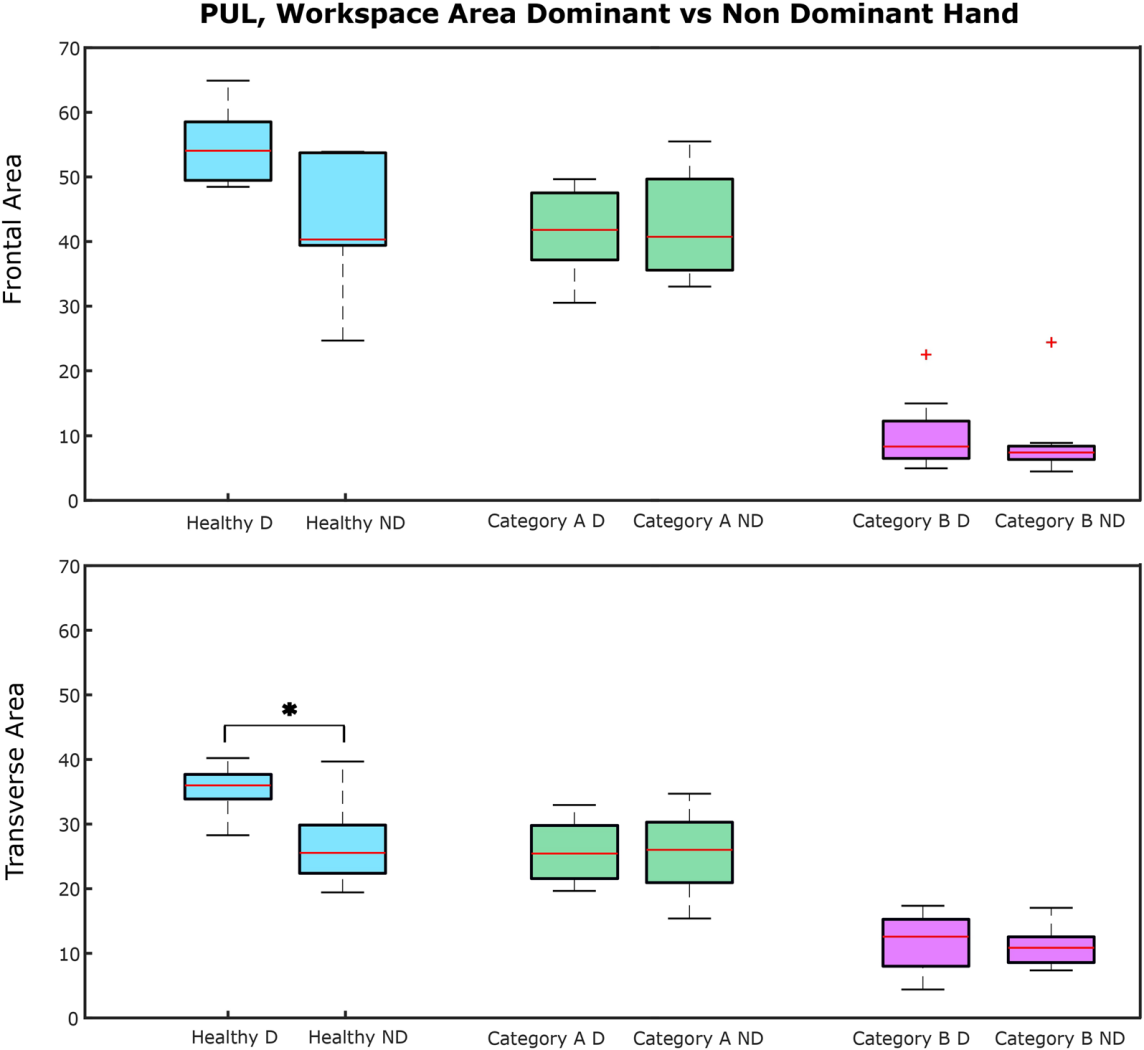
Boxplots of the workspace area of children with DMD comparing the dominant (D) and non-dominant (ND) side

**Fig. 6:**
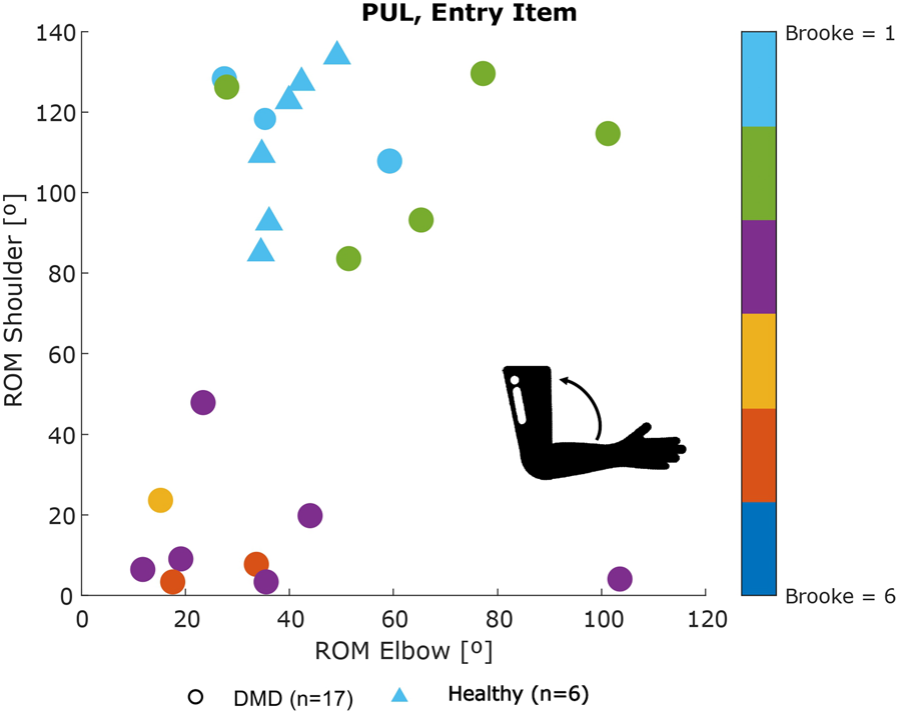
Scatter plot of shoulder ROM versus elbow ROM for the Entry item of the PUL

### Kinematic analysis of participants with DMD

As seen in Fig. 3, strong correlations were found between clinical score and **workspace area** in the frontal plane ($\rho = 0.94$, p < 0.01) and in the transverse plane ($\rho = 0.90$, p < 0.01), as well as **workspace volume** ($\rho = 0.92$, p < 0.01) and shoulder-elbow **curve efficiency** ($\rho = 0.84$, p < 0.01). To provide further insight, we evaluated the **workspace area** reached for each item. In Fig. 4, we report the items that showed statically significant differences between categories. Interestingly, statistically significant differences were found for the entry item, the segmented recording, and the full recording, in both the frontal and transverse areas, between healthy group and Category A, and between Category A and Category B. Additionally, statistically significant differences were found between the workspace areas of the segmented recording and the full recording for all the categories. In Fig. 5, we also evaluated the **workspace area** for the non-dominant hand. In the frontal plane, no significant statistical differences were found for any categories. In the transverse plane, a statistically significant difference was found only for the healthy group. The median values of these metrics can be found in the Appendix B. Figure 6 shows the analyses of the **ROM** of shoulder and elbow. To evaluate the compensatory strategies used by the children to complete the items, we analyzed the shoulder and elbow ROM for the entry item. This item was chosen because it is used to assign the Brooke score and determines the items that will be asked of the patient during the clinical assessment. As expected, two main groups were identified: children who can abduct the arms and those who cannot. The first group corresponds to healthy children and children in Category A, while the second group corresponds to children in Category B.

Table 3 shows an overview of the computed kinematic metrics (median (interquartile range (IQR))) of participants with DMD, organized into categories. In general, a decrease in parameter values can be discerned as Brooke score increases, which corresponds to a reduced motor function. Between healthy participants and participants with DMD, as well as between Category A and Category B, the majority of metrics demonstrate statistically significant differences.

**Table 3:** Kinematic metrics, median (IQR), of DMD patients and healthy children per category

	Healthy	DMD	
Variable (unit)		Category A (Brooke $\le$ 2)	Category B (Brooke >2)
Number of participants in Category	6	8	9
Ages (y)^3^	10.50 (6.00)	11.50 (3.00)	15.00 (2.00)
Areas Frontal Plane (%)^1,2,3,4^	54.05 (10.89)	41.97 (12.67)	8.53 (7.09)
Areas Transverse Plane (%)^1,2,3,4^	32.36 (5.83)	24.51 (9.78)	9.19 (8.12)
Volumes (%)^1,2,3,4^	34.92 (10.44)	25.92 (10.92)	3.54 (4.13)
Curve Efficiency (deg/sample)^1,3,4^	0.54 (0.14)	0.48 (0.19)	0.19 (0.13)
Shoulder ROM (Pl. Elev.) (deg)^1,3,4^	242.09 (121.01)	147.22 (79.12)	112.70 (53.93)
Shoulder ROM (Elev.) (deg)^1,3,4^	127.29 (22.69)	125.72 (26.36)	45.42 (42.17)
Shoulder ROM (Ax. Rot.) (deg)	178.27 (137.24)	101.63 (90.04)	88.12 (114.14)
Elbow ROM (Flex./Ext.) (deg)^3,4^	133.92 (26.90)	145.70 (49.16)	97.66 (41.22)
Elbow ROM (Abd./Add.) (deg)	74.98 (16.68)	80.32 (23.27)	63.92 (18.30)
Elbow ROM (Pro./Sup.) (deg)^1,3^	145.48 (26.31)	109.23 (51.47)	84.32 (50.18)
Max. Linear Velocity Hand (cm/s)^3,4^	122.16 (28.47)	126.93 (37.82)	71.71 (24.86)
Clinical Score^1,2,3,4^	42.00 (0.00)	37.00 (6.00)	19.00 (5.00)

Superscripts 1,2,3, and 4 represent a statistically significant difference between: ^1^ healthy and DMD, ^2^ healthy and Category A, ^3^ healthy and Category B, and ^4^Category A and Category B

### Kinematic analysis of participants with SMA

**Fig. 7:**
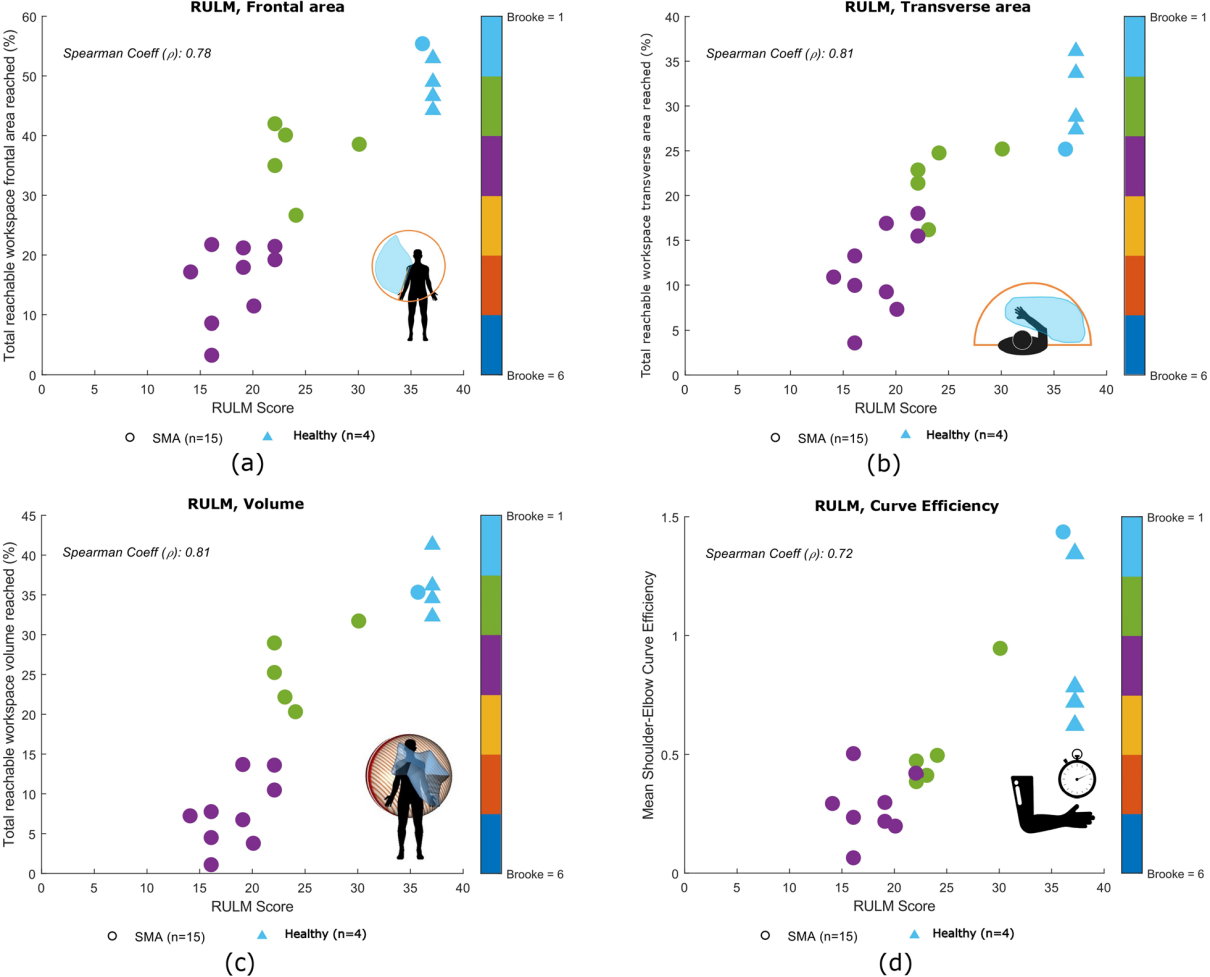
Correlation plots for children with SMA (RULM clinical scale): **a** correlation between clinical score and workspace area in the frontal plane, **b** correlation between clinical score and workspace area in the transverse or horizontal plane, **c** correlation between the clinical score and workspace volume **d** correlation between the clinical score and curve efficiency. Spearman correlation coefficient $\rho$ between the metric and clinical score is reported. Healthy children are not included in the correlation analysis

**Fig. 8:**
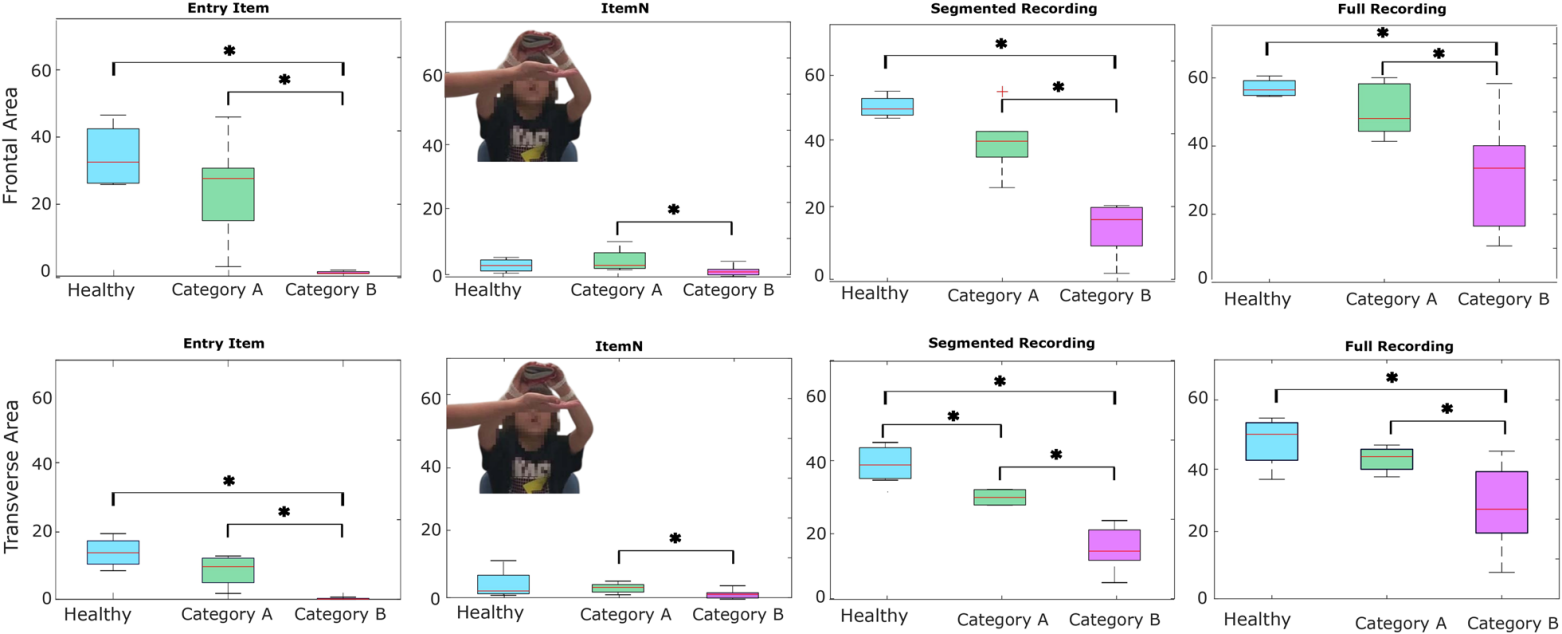
Boxplots of the workspace area of children with SMA in the frontal plane (upper row) and transverse plane (lower row) for the entry item, item N, for all the items after the annotation and without annotation

As can be observed in Fig. 7, a strong correlation was found for the **workspace area** in the frontal and transverse planes ($\rho =0.78$, p < 0.001 and $\rho =0.81$, p < 0.001, respectively), as well as for the **workspace volume** ($\rho =0.81$, p < 0.001). A fair correlation was found for the **curve efficiency** ($\rho =0.72$, p < 0.001). In Fig. 8 it is possible to see that statistically significant differences were identified across the categories (Healthy, Category A and Category B) for the entry item, Item N (Bring 500 g sand weight from lap to table or eye level), for the both segmented recording and for the full recording. See Appendix A for more details regarding the description and scoring of item N.

**Fig. 9:**
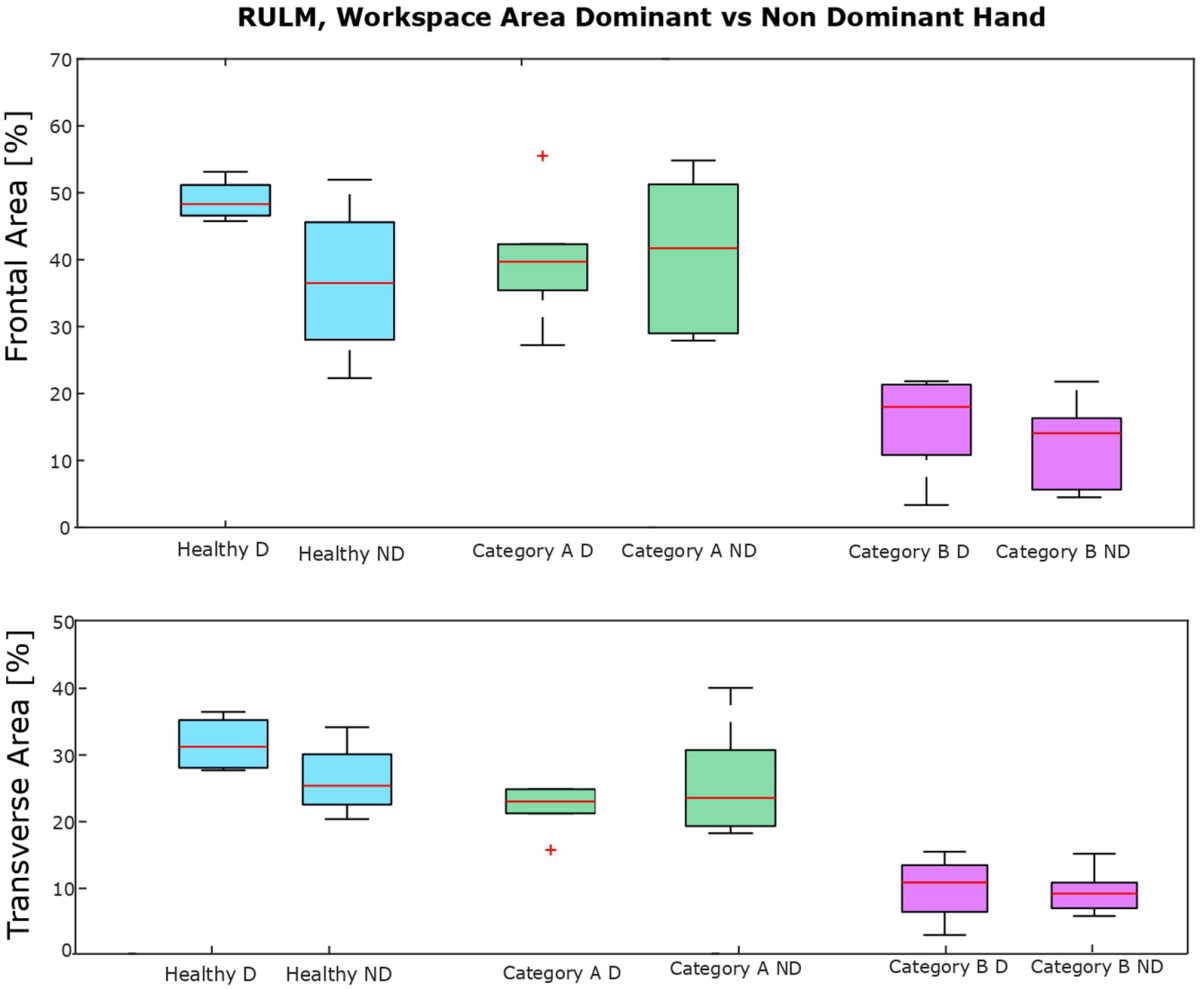
Boxplots of the workspace area of children with SMA comparing the dominant (D) and non-dominant (ND) side

**Fig. 10:**
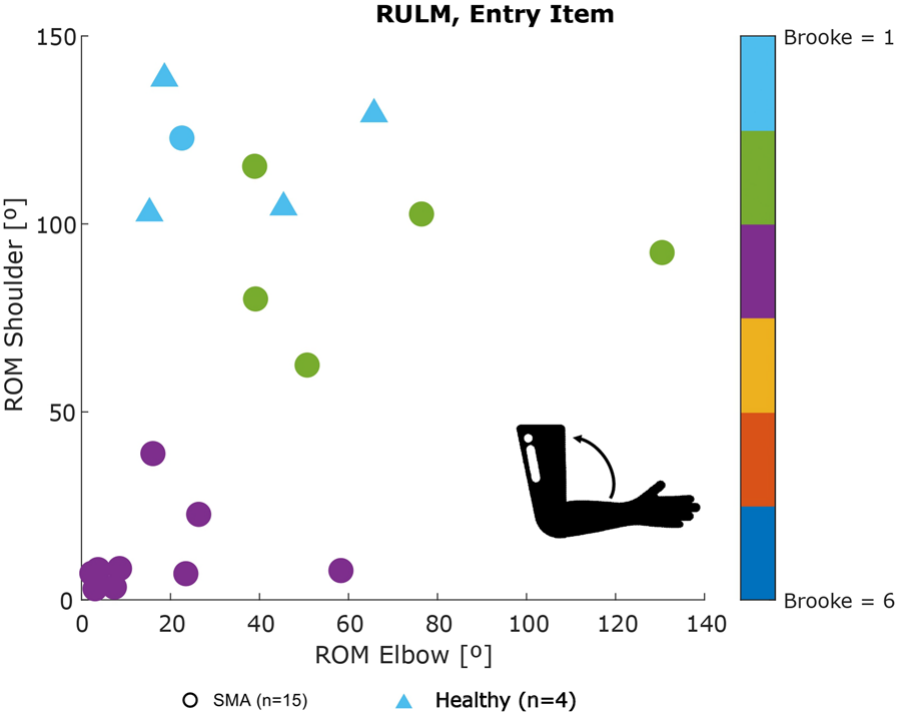
Scatter plot of shoulder ROM versus elbow ROM for the Entry item of the RULM

Statistically significant differences were observed within all categories between **workspace area** of the segmented and full recording. In Fig. 9 we report the analysis of the workspace area for the non-dominant side.No significant statistical differences were found for any categories. The median values of these metrics can be found in the Appendix B. In Fig. 10, to quantify the compensation performed by the children, during the execution of the entry item, we report the comparison between the **ROM** of the shoulder and the elbow. In Table 4, it is possible to observe a decrease in the metrics analyzed, from healthy children to those with SMA, with further reductions observed as Brooke scores increase. This tendency is clear in particular for the **workspace area** in the frontal and transverse planes, the **volume** and the **curve efficiency**. These results are confirmed by statistical analysis.

**Table 4:** Kinematic metrics, median (IQR), of SMA patients and healthy children per category

	Heathy	SMA	
Variable (unit)		Category A (Brooke $\le$ 2)	Category B (Brooke $>2$)
Number of participants in Category	4	6	9
Ages (y)^4^	8.00 (7.50)	8.50 (5.00)	12.00 (3.00)
Areas Frontal Plane (%)^1,2,3,4^	48.31 (5.95)	39.49 (12.43)	18.10 (11.29)
Areas Transverse Plane (%)^1,2,3,4^	31.02 (7.95)	23.48 (4.93)	11.00 (7.91)
Volumes (%)^1,2,3,4^	35.01 (6.91)	27.23 (10.92)	7.36 (7.89)
Curve Efficiency (deg/frame)^1,3^	0.75 (0.55)	0.49 (0.66)	0.29 (0.27)
Shoulder ROM (Pl. Elev.) (deg)	143.75 (54.16)	148.04 (30.88)	169.13 (137.07)
Shoulder ROM (Elev.) (deg)^1,3,4^	132.76 (28.00)	120.51 (36.84)	47.54 (67.93)
Shoulder ROM (Ax. Rot.) (deg)	124.30 (65.40)	139.04 (42.11)	138.85 (104.12)
Elbow ROM (Flex.)(deg)^4^	155.39 (18.11)	170.25 (47.36)	146.72 (60.20)
Elbow ROM (Abd./Add.) (deg)	68.90 (27.88)	97.05 (19.74)	88.84 (52.67)
Elbow ROM (Pron./Sup.) (deg)^4^	140.85 (32.26)	141.27 (54.07)	122.19 (43.28)
Max. Linear Velocity Hand (cm/s)^1,3^	155.64 (52.48)	120.56 (71.55)	110.08 (66.64)
Clinical Score^1,2,3,4^	37.00 (0.00)	23.50 (8.00)	17.00 (4.00)

Superscripts 1,2,3 and 4 represent a statistically significant difference between: ^1^ healthy and SMA, ^2^ healthy and Category A, ^3^ healthy and Category B, and ^4^Category A and Category B

## Discussion

The purpose of this study was to assess the effectiveness of using an IMU-based system to objectively quantify the motor performance of the upper limb in children with neuromuscular diseases. For this reason, in this section, we will discuss the kinematic metrics listed above for both groups of children with DMD and SMA. The analysis will cover the segmented recording and the full recording, and specific items that are important for disease assessment.

A good correlation between the kinematic metrics analyzed and results of clinical assessment was found. Additionally, these findings supported the hypothesis that kinematic analysis performed with the system is effective for an accurate evaluation and provides additional information not available from traditional clinical evaluation. Thus, these results show promise that these metrics can be used for an objective evaluation of the diseases.

Regarding the workspace area, for the DMD group, a strong correlation was found between this metric and the PUL score. This result was expected, as it indicates that children in Category A can reach a larger area and are able to control their upper limbs. This ability progressively decreases with the severity of the disease. Han et al. also reported significant differences in reachable workspace areas between DMD patients with different Brooke scores. However, in such case the authors analyzed the three dimensional area of the sphere that the arm reaches in extension, rather than its projection onto the frontal and transverse planes [31]. We decided to analyze the workspace area projected on two distinct planes to give more insight into each separate plane. For the SMA group, children with a similar clinical score showed variability in workspace areas, suggesting that this metric may not be adequate to assess motor function in patients with SMA. Conversely, this finding might suggest that this metric offers additional insight beyond clinical scoring alone providing a finer-grained evaluation compared to the broader one obtained through clinical assessment.

When evaluating the workspace area of each item separately, the entry item was the one that contributed the most to the total area, for both DMD and SMA group. This result was expected because the entry item gives a general overview of the child’s motor status. Interestingly, statistically significant differences were found for item N for the SMA group. A possible explanation might be related to the importance of the shoulder, to perform this item. The shoulder is the first joint to be affected by the disease and the accurate evaluation of this item might help the clinician to recognize earlier the decrease of the motor functions. For both DMD and SMA, a statistically significant increase was found between the workspace of the segmented recording and of the full recording, suggesting that patients achieve a larger workspace area than the one assessed during the clinical scale. This suggests that the clinical assessment may be underestimating the patients’ actual range of movement, as they can achieve a greater range in a broader context than what is measured in the standard scale.

In the healthy group, a decrease in workspace area between the dominant and non-dominant sides was found, likely due to their preference for using the dominant hand. This trend was not clear in children with neuromuscular diseases, who might be used to employing both arms.

A strong correlation was identified between the clinical score and the workspace volume reached for both groups, aligning with previous studies [16]. Again, this finding is in line with our hypothesis, since the volume that can be reached relies heavily on the motor function of the upper extremity.

The results of the curve efficiency have to be interpreted with caution because this metric is influence by the speed at which the item was performed and the children were not instructed to perform the scale at any specific speed. In case of children with DMD, a decrease was noted as Brooke scores increases, indicating that efficiency of the interjoint coordination decrease with the progression of the disease. For children with SMA, no statistically significant differences were found between Category A and Category B. These findings suggest that this metric may be inadequate for evaluating children with SMA due to its reliance on velocity. Alternatively, it is possible that children with SMA experience rapid disease progression at the onset, resulting in no clear distinction between children in Category A and in Category B. It is worth underlining that the two children (one with SMA and one healthy child) who achieved high efficiency performed the task at a fast pace. This had an influence on the efficiency, positively influenced by the velocity. Schwarz et al. [29] also identified a strong correlation between curve efficiency and upper limb function, although their study was conducted with post-stroke individuals.

Generally, a decrease in ROM of upper extremity joints for children with neuromuscular diseases is observed, as previously noted in [15, 32]. In a few cases, we observed a higher ROM in children in Category A than healthy children. This finding may be associated with compensatory movements performed by children with NMDs. For instance, children with NMDs tend to flex the elbow to achieve shoulder elevation. For children with SMA, we found statistically significant difference between Categories A and B for the elbow flexion, while Janssen et al. did not [15]. However, Janssen et al. focused on a pure elbow flexion exercise, while our study involves a comprehensive evaluation of elbow kinematics during the clinical evaluation. Chen et al. did not find differences in shoulder and elbow ROM between children with SMA and healthy children either, while they were performing tasks from daily activities in a simulated home environment [17]. However, the authors analyzed children with SMA type III that is a milder form with respect to SMA type II. Among the children with SMA analyzed in this study, just three children were diagnosed with SMA type III while the others were diagnosed with type II.

When analyzing shoulder elevation and the elbow flexion ROM for the entry item, it was possible to distinguish the children who could abduct their arms (upper part of the plot) and those could not (lower part of the plot), for both DMD and SMA groups. Children with Brooke score 1 were able to ’abduct both arms in full extension’, while children with Brooke 2 compensated by flexing the elbow. Interestingly, one patient with Brooke score 2 had similar shoulder and elbow ROM to a patient with Brooke score 1. This discrepancy suggests that sensor data might provide a more objective analysis than a physiotherapist’s evaluation. So, we requested two different clinicians to re-evaluate this item for this patient based on the video recording and no clear consensus was reached.

Regarding the linear velocity of the upper extremity, it has to be noted that the children were not asked to perform the items at any specific speed. Nevertheless, it was possible to identify a decrease of the speed for the DMD group with the progression of the disease, likely due to muscle weakness[16]. For SMA, differences between healthy and SMA groups were noted, but not within the SMA categories, possibly due to the absence of speed-related task instructions. While this study provides encouraging insights, some areas deserve further consideration. First, a broader sample size, particularly in SMA cases, could allow for a more comprehensive analysis across disease severity levels. Specifically, future research should include a broader range of patients with Brooke score 1 and Brooke score > 3. Nevertheless, we were able to distinguish two main categories (Category A and Category B) that allow classification according to the disorder severity. We used a kinematic model considering only rotational degree of freedom. Future work should also implement translational degrees of freedom, particularly for the shoulder. The shoulder is the first joint of the upper limb affected by DMD and SMA and a deeper understanding of its kinematics might beneficial for patients. In this study, we focused on kinematic analysis only, while muscle strength represents an important factor for the upper limb motor performance. Hence, incorporating muscle strength measurements alongside kinematic data could give a more complete view of upper limb performance. This combination would help differentiate between movement limitations due to joint stiffness versus muscle weakness, allowing for a more targeted approach in intervention planning. Further, in the presented study, the IMU-based system was only used in a controlled environment and in combination with video footage. Future research should explore its applicability in uncontrolled settings, such as at home or school environments. Additionally, assessing disease progression in daily life represents a promising direction for future research. Finally, in order to reduce the number of sensors and the burden for the children, future research could remove the sensors on the hand, as they are the least influential on the most important metrics, such as workspace area, volume, and curve efficiency.

## Conclusion

This study demonstrates the potential of using an IMU-based system to provide accurate, and quantitative assessment of upper limb motor function in children with neuromuscular diseases. The results reveal that kinematic metrics, such as normalized workspace area and volume, along with curve efficiency, strongly correlate with clinical scores and reflect the progressive motor impairment typical of DMD and SMA. Workspace area, both in the frontal and transverse planes, was particularly effective in distinguishing motor function across disease severity levels, adding valuable insight beyond traditional clinical assessment. The volume reached and shoulder-elbow curve efficiency also provided reliable indicators of motor function, with clear decreases observed as disease severity increased. Additionally, ROM can assist clinicians in quantifying compensatory strategies adopted by the patients to complete the items, such as elbow flexion when shoulder elevation was limited. Although linear velocity exhibited a weaker correlation with clinical scores than the other analyzed metrics, we observed a decline in speed with DMD progression; This finding suggests that linear velocity may serve as a valuable parameter for tracking disease evaluation. Overall, this IMU-based system offers clinicians a reliable, and objective means to assess upper limb functionality in a clinical setting; complementing traditional evaluations and potentially enhancing individualized treatment plans. Future research should aim to validate these findings across larger cohorts and explore practical applications for continuous home monitoring, focusing on early intervention and management of neuromuscular disease progression.

## Data Availability

The datasets generated during the current study are available from the corresponding author upon reasonable request.

## Appendix A items mentioned within the text

This section reports additional details of the items mentioned within the text (Table 5).

**Table 5:** Detailed description of the items mentioned or analyzed within the text

Item	Description	Scale	Score
Item 1	Shoulder abduction both arms above head	PUL	According to the compensation performed, the score varies between 0 and 2, going from ’unable’ to ’Can abduct both arms simultaneously elbows in full extension until the elbows approximate the ears’
Item 2	Raise both arms to shoulder height	PUL	According to the compensation performed, the score varies between 0 and 2, going from ’unable’ to ’Can raise both arms to shoulder height simultaneously without compensation’
Item O	Bring both arms above head	RULM	According to the compensation performed, the score varies between 0 and 2, going from ’unable’ to ’Can abduct both arms simultaneously-elbows in extension in a full circle until they touch above the head’
Item N	Bring 500g sand weight from lap to table or eye level	RULM	According to the compensation performed, the score varies between 0 and 2, where 0 represents ’unable’, 1 represents ’brings weight onto table using two hands’ and 2 presents’ brings weight at eye level

## Appendix B results-additional information

This section reports the median (IQR) values of the items that showed statistically significant difference among groups (healthy, Category A and Category B) and within groups (Tabels 6, 7, 8 and 9).

**Table 6:** Median (IQR) of DMD group of the item, segmented recording and full recording significantly statistically different among healthy participants, Category A and Category B

PUL Frontal plane	Healthy	Category A	Category B
$\text {Entry item}^{\dagger ,\ddagger }$	38.1 (18.2)	30.7 (13.1)	0.5 (0.5)
$\text {Segmented recording}^{\dagger ,\ddagger }$	54.1 (10.8)	42.0 (12.7)	8.5 (7.1)
$\text {Full recording}^{\dagger ,\ddagger }$	61.7 (11.7)	50.2 (10.7)	21.7 (18.2)
**PUL Transverse Plane**	**Healthy**	**Category A**	**Category B**
$\text {Entry Item}^{\dagger ,\ddagger }$	10.7 (8.1)	12.6 (12.5)	0.3 (0.4)
$\text {Segmented recording}^{\dagger ,\ddagger }$	32.4 (5.8)	24.5 (9.7)	9.2 (8.1)
$\text {Full recording}^{\dagger ,\ddagger }$	39.1 (11.3)	32.1 (14.6)	17.8 (14.4)

*symbol represents significance between healthy group and Category A, $\dagger$ represents significance between healthy group and Category B, $\ddagger$ represents significance between Category A and Category B

**Table 7:** Median (IQR) of SMA group of the item, segmented recording and full recording significantly statistically different among healthy participants, Category A and Category B

RULM Frontal plane	Healthy	Category A	Category B
$\text {Entry Item}^{\dagger ,\ddagger }$	29.9 (15.7)	25.9 (21.2)	0.1 (0.5)
Item N $^{\ddagger }$	2.9 (3.8)	3.2(5.2)	1.4 (1.9)
Segmented Recording$^{\dagger ,\ddagger }$	48.3 (5.9)	39.5 (12.4)	18.1(11.3)
Full Recording$^{\dagger ,\ddagger }$	56.5 (5.2)	51.2 (11.6)	32.9 (18.7)
**RULM Transverse Plane**	**Healthy**	**Category A**	**Category B**
Entry Item$^{\dagger ,\ddagger }$	11.7 (7.6)	8.5 (6.4)	0.7 (0.2)
Item N $^{\ddagger }$	1.9 (5.8)	2.7 (2.4)	1.3(1.3)
Segmented Recording *$^{\dagger ,\ddagger }$	31.0 (7.9)	23.5(4.9)	11.0 (7.9)
$\text {Full Recording}^{\dagger ,\ddagger }$	41.4 (12.4)	35.7(9.0)	24.3(13.0)

*symbol represents significance between healthy group and Category A, $\dagger$ represents significance between healthy group and Category B, $\ddagger$ represents significance between Category A and Category B

**Table 8:** Median (IQR) of DMD group for the dominant and non dominant side significantly statistically different among healthy participants, Category A and Category B

PUL Frontal plane	Dominant	Non dominant
Healthy	54.1 (10.8)	40.3 (18.1)
Category A	42.0 (12.7)	41.0 (14.9)
Category B	8.5 (7.1)	7.6 (2.7)
**PUL Transverse Plane**	**Dominant**	**Non Dominant**
Healthy *	32.4 (5.8)	22.0 (10.7)
Category A	24.5 (9.8)	25.1 (10.5)
Category B	9.2 (8.1)	7.5 (4.9)

*symbol represents significance within the group

**Table 9:** Median (IQR) of SMA group for the dominant and non dominant side significantly statistically different among healthy participants, Category A and Category B

RULM Frontal plane	Dominant	Non dominant
Healthy	48.3 (5.9)	36.5 (23.6)
Category A	39.5 (12.4)	41.5 (23.8)
Category B	18.1 (11.4)	14.1 (10.97)
**RULM Transverse Plane**	**Dominant**	**Non Dominant**
Healthy	31.0 (7.9)	25.2 (10.7)
Category A	23.5 (4.9)	24.0(13.8)
Category B	11.0 (7.9)	12.0(9.8)

*symbol represents significance within the group

## Abbreviations

IMU: Inertial Measurement Units
DMD: Duchenne Muscular Dystrophy
SMA: Spinal Muscular Atrophy
$$\rho$$: Spearman Coefficient
NMDs: Neuromuscular diseases
PUL: Performance Upper Limb
RULM: Revised Upper Limb Module
ROM: Range of Motion
